# Quantitative proteomics reveals the effect of protein glycosylation in soybean root under flooding stress

**DOI:** 10.3389/fpls.2014.00627

**Published:** 2014-11-18

**Authors:** Ghazala Mustafa, Setsuko Komatsu

**Affiliations:** ^1^Graduate School of Life and Environmental Science, University of TsukubaTsukuba, Japan; ^2^National Institute of Crop Science, National Agriculture and Food Research OrganizationTsukuba, Japan

**Keywords:** soybean, flooding stress, root, proteomics, glycoproteins

## Abstract

Flooding stress has a negative impact on soybean cultivation because it severely impairs growth and development. To understand the flooding responsive mechanism in early stage soybeans, a glycoproteomic technique was used. Two-day-old soybeans were treated with flooding for 2 days and roots were collected. Globally, the accumulation level of glycoproteins, as revealed by cross-reaction with concanavalin A decreased by 2 days of flooding stress. Glycoproteins were enriched from total protein extracts using concanavalin A lectin resin and analyzed using a gel-free proteomic technique. One-hundred eleven and 69 glycoproteins were identified without and with 2 days of flooding stress, respectively. Functional categorization of these identified glycoproteins indicated that the accumulation level of proteins related to protein degradation, cell wall, and glycolysis increased, while stress-related proteins decreased under flooding stress. Also the accumulation level of glycoproteins localized in the secretory pathway decreased under flooding stress. Out of 23 common glycoproteins between control and flooding conditions, peroxidases and glycosyl hydrolases were decreased by 2 days of flooding stress. mRNA expression levels of proteins in the endoplasmic reticulum and N-glycosylation related proteins were downregulated by flooding stress. These results suggest that flooding might negatively affect the process of N-glycosylation of proteins related to stress and protein degradation; however glycoproteins involved in glycolysis are activated.

## Introduction

Climate change is potentially the greatest threat to biodiversity (Eigenbrod et al., 2014). The industrial revolution has resulted in elevated levels of carbon dioxide and other greenhouse gases that induce global warming and change precipitation patterns (Hao et al., 2010). Increasing climatological extremes lead to catastrophic loss of crop productivity (Bita and Greats, 2013). In these changing conditions, plants are under the effects of various abiotic stresses like drought (Manavalan et al., 2009), salinity (Parvaiz and Satyawati, 2008), cold (Beck et al., 2004, 2007), and high temperature (Bita and Greats, 2013). Flooding has devastating effects on crop growth and ultimately causes a reduction in crop production (Normile, 2008).

Soybean is an important legume crop due to its high protein content. Soybean is susceptible to flooding stress (Hou and Thseng, 1991), a major problem that affects its growth and yield around the world. The grain yield of this crop is particularly affected by this stress, notably during seed germination and early vegetative stages (Githiri et al., 2006). Early exposure of soybean plants to flooding stress causes severe damage due to rapid imbibition of water by the cotyledons and destruction to the root systems (Nakayama et al., 2004). Its yield was estimated to be reduced to 25% due to flooding injuries in Asia, North America, and other regions of the world where soybean is rotated with rice in paddy fields. Oosterhuis et al. (1990) reported a reduction in soybean yield of 17–43% at the vegetative stage and 50–56% at the reproductive stage due to flooding stress. This stress leads to a shift to alternative pathways of energy generation. The shortage of oxygen under flooding stress results in a shift from aerobic to anaerobic respiration. A low diffusion rate of oxygen under flooding stress is a limiting factor for plant survival, and most plants die under limited oxygen supply (Voesenek et al., 2006).

The process of glycosylation is a complicated and highly important post-translational modification occurring in natural proteins. Protein glycosylation results from the covalent linkage of an oligosaccharide side chain to a protein moiety (Spiro, 2002). The vast majority of eukaryotic proteins are glycosylated. This protein modification plays an important role in protein folding, interaction, stability, and mobility, as well as in signal transduction (Roth et al., 2012). Glycosylated proteins are involved in many physiological functions and biological pathways (Pan et al., 2011; Ruiz-May et al., 2012). In plants, N-linked glycans have various roles including the prevention of proteolytic degradation, induction of correct folding, and biological activity of a protein. Along with this, they also contain targeting information and are involved in the protein recognition or cell-cell adhesion processes (Rayon et al., 1998). In animals, oligosaccharide-side chains act as targeting signal for lysosomal glycoproteins (Sly and Fischer, 1982). In plants, complex N-glycans confer important functions to secreted/secretory glycoproteins, such as protection of root growth from osmotic stress (von Schaewen et al., 2008).

Glycosylation is of two main types: N-glycosylation and O-glycosylation. N-linked glycan biosynthesis starts at the cytosolic face of the endoplasmic reticulum (ER) where two N-acetylglucosamine and five mannose residues are added sugar by sugar onto a dolichol carrier (Kornfeld and Kornfeld, 1985). In eukaryotes, several protein modifications, including the glycosylation reaction, occur in the ER (Abeijon and Hirscnberg, 1992). The ER is also responsible for various other cellular functions like protein folding, degradation, protein synthesis, lipid synthesis, and transfer (Coe and Michalak, 2010). Flooding has severe effects on ER function due to changes in the levels of calnexin, heat shock protein 70, and luminal binding protein (Nanjo et al., 2010). But the specific nature of these effects is still not clear. It has been reported that flooding stress causes damage to the ER and to the process of glycosylation (Komatsu et al., 2012). However, the relationships and interactions between different glycosylated proteins have not been fully characterized.

Because of its importance to cell functions, a comprehensive understanding of glycoproteins is of great importance to elaborate their roles and to understand plant responses to flooding stress. In this study, to understand the early responses of soybean roots to flooding stress, glycoproteins were analyzed from young soybean roots. For this purpose, the concanavalin A (ConA) lectin affinity method was used to target the glycoproteins (Yang and Hancock, 2004), and gel-free proteomics with liquid chromatography (LC) mass spectrometry (MS) was performed. ConA affinity chromatography was previously used to characterize the glycoproteome of tomato fruit (Catala et al., 2011) and in proteomic characterization of plant secreted proteins (Minic et al., 2007; Ligat et al., 2011). Furthermore, the mRNA expression levels of proteins in the ER and N-glycosylation related proteins were analyzed using quantitative reverse transcription-polymerase chain reaction (qRT-PCR).

## Materials and methods

### Plant material

Seeds of soybean (*Glycine max* L. cv. Enrei) were sterilized with 1% sodium hypochlorite solution, rinsed in water, and sown on 500 mL silica sand with 150 mL water in a plastic case (180 × 140 × 45 mm). Soybean was grown in a growth chamber illuminated with white fluorescent light (160 μmol m^−2^ s^−1^, 16 h light period/day) at 25°C and 70% relative humidity. For ConA blotting, 2-day-old soybeans were flooded with water (Komatsu et al., 2010), for 1–4 days. For proteomics, 2-day-old soybeans were flooded for 2 days. For qRT-PCR, 2-day-old soybeans were flooded for 1 and 2 days. After treatments, roots were collected. Untreated plants were used as controls. Three independent experiments were performed as biological replicates for all experiments.

### Protein extraction

A portion (500 mg) of samples was homogenized on ice using a mortar and pestle in buffer containing 20 mM HEPES (pH 7.5), 150 mM NaCl, 1% Nonidet P-40, 0.25% sodium deoxycholate, and 10% glycerol. The homogenate was centrifuged at 20,000 × g for 10 min at 4°C. The supernatant was collected and centrifuged at 20,000 × g for 10 min at 4°C again. The supernatant was used as the total protein extract. Protein concentration was determined using the Bradford method (Bradford, 1976) with bovine serum albumin as the standard. For MS analysis, this total protein extract was purified as described below in the paragraph “Preparation of Proteins for Mass Spectrometry.” For SDS-PAGE, 2x SDS sample buffer containing 120 mM Tris-HCl (pH 6.8), 4% SDS, 20% glycerol, and 10% 2-mercaptoethanol was added in equal volume.

### Glycoprotein enrichment

The extracted proteins were submitted to glycoprotein enrichment by using the glycoprotein isolation kit-ConA (Thermo Fisher Scientific, San Jose, CA, USA). All steps were performed at 25°C. ConA resin was added to the spin column and centrifuged at 1000 × g. Resin was rinsed three times with binding buffer provided in the kit. Before being applied to the ConA resin column, the protein samples were equilibrated with binding buffer. After 10 min of mixing, the resin was centrifuged at 1000 × g. Resin was washed four times with binding buffer and then glycoproteins were eluted in SDS sample buffer containing 60 mM Tris-HCl (pH 6.8), 2% SDS, 10% glycerol, and 5% 2-mercaptoethanol. Protein concentration was determined using the Pierce 660 nm Protein Assay Reagent (Thermo Fisher Scientific) with bovine serum albumin as the standard.

### SDS-polyacrylamide gel electrophoresis

The proteins resulting from the total protein extract or from glycoprotein enrichment were separated by 17% SDS-polyacrylamide gel electrophoresis. The electrophoresis was performed at a constant current of 20 mA. After electrophoresis, the gels were stained for 1 h with Coomassie brilliant blue (CBB) (PhastGel™ Blue R; GE Healthcare, Piscataway, NJ, USA) containing 30% methanol and 10% acetic acid, and then destained for 2 h in destaining solution containing 36% methanol and 10% acetic acid.

### Concanavalin a blotting

For immunoblot analysis, proteins were separated by 17% SDS-PAGE. After separation, proteins were transferred to a polyvinylidene difluoride membrane using a semidry transfer blotter. Blotted membrane was blocked overnight at 4°C in a buffer containing 20 mM Tris-HCl (pH 7.5), 500 mM NaCl, and 5% nonfat milk (skim milk; Difco, Sparks, MD, USA) and incubated with a 1:2000 dilution of peroxidase-ConA antibody (Seikagaku, Tokyo, Japan), for 1 h at 25°C. Glycoproteins were detected using an ECL plus Western blotting detection kit (GE Healthcare) following the manufacturer's protocol, and visualized by a luminescent image analyzer (Las-3000; Fujifilm, Tokyo, Japan). The relative band intensities were calculated using ImageJ software (http://imagej.nih.gov/ij/).

### Preparation of proteins for mass spectrometry

For MS analysis, proteins (100 μg) were purified by phase separation in the organic layer. In 100 μL protein sample, 400 μL methanol was added, and the resulting solution was mixed. After this, 100 μL chloroform was added and mixed by vortexing. Then 300 μL water was added to induce phase separation, mixed, and then centrifuged at 20,000 × g for 10 min. The upper aqueous layer was discarded and 300 μL methanol was added to the organic phase. The samples were centrifuged at 20,000 × g for 10 min. The resulting supernatant was discarded and the pellet was allowed to dry at 25°C. The dried pellets were resuspended in 50 mM ammonium bicarbonate and then reduced with 0.25 M dithiothreitol for 1 h at 56°C and alkylated with 0.3 M iodoacetamide for 1 h at 37°C in the dark. Alkylated proteins were digested with trypsin and lysyl endopeptidase (sequencing grade; Wako, Osaka, Japan) at 1:100 enzyme/protein concentration at 37°C for 16 h. The resulting tryptic peptides were acidified with 10 μL of 20% formic acid to pH < 3, desalted with a C18-pipette tip (NikkyoTechnos, Tokyo, Japan), and subjected to nanoLC MS/MS.

### Data acquisition by mass spectrometry

Using an Ultimate 3000 nanoLC system (Dionex, Germering, Germany), peptides in 0.1% formic acid were loaded onto a C18 PepMap trap column (300 μm ID × 5 mm, Dionex). The peptides were eluted from the trap column and were separated using 0.1% formic acid in acetonitrile at a flow rate of 200 nL/min on a C18 Tip column (75 μm 1D × 120 mm, NTTC-360/75-3, nanoLC capillary column (Nikkyo Technos) with a spray voltage of 1.5 kV. Peptides were analyzed on a nanospray LTQ XL Orbitrap MS (Thermo Fisher Scientific) operated in data-dependent acquisition mode with the installed Xcalibur software (version 2.0.7; Thermo Fisher Scientific). Elution was performed with a linear acetonitrile gradient (15–40% in 115 min) in 0.1% formic acid. Full-scan mass spectra were acquired in the Orbitrap MS over a mass range of 400–15,000 *m/z* with a resolution of 30,000. A lock mass function was used to obtain high mass accuracy (Olsen et al., 2005). The top 10 most intense precursor ions were selected for collision-induced fragmentation in the linear ion trap at normalized collision energy of 35%. Dynamic exclusion was employed within 90 s (Zhang et al., 2009) to prevent repetitive selection of peptides.

### Identification of proteins obtained by mass spectrometry

Identification of proteins was performed by the MASCOT search engine (version 2.4.1) (Matrix Science, London, UK) and Proteome Discoverer (version 1.4.0.288; Thermo Fischer Scientific) against a soybean peptide database (54,175 sequences) (Phytozome version 9.0, http://www.phytozome.net/soybean) (Schmutz et al., 2010). Parameters used in MASCOT searches were as follows: Carbamidomethylation of cysteine was set as a fixed modification, and oxidation of methionine was set as a variable modification. Trypsin was specified as the proteolytic enzyme and one missed cleavage was allowed. Peptide mass tolerance was set at 5 ppm, fragment mass tolerance was set at 0.8 Da, and peptide charge was set at +2, +3, and +4. An automatic decoy database search was also performed. MASCOT results were filtered with MASCOT percolator to improve accuracy and sensitivity in the peptide identification (Brosch et al., 2009). False discovery rates for peptide identification of all searches were less than 1.0%. Peptides with a more than 13 peptide probability and percolator *q*-value 0.01 were used for protein identification. The MASCOT results generated msf files were used for SIEVE (version 2.0; Thermo Fisher Scientific) analysis.

To compare protein and peptide contents between different groups, extracted ion chromatograms (XIC) based comparison approach was used in the SIEVE software. For differential analysis of the relative abundance of peptides and proteins between the control and treatment groups, the commercial label-free quantification package SIEVE was used. The chromatographic peaks obtained from MS were aligned and the peptide peaks were detected as frames using the following settings: frame time width (5 min); frame *m/z* width (10 ppm); produce frames on all parent ions subjected to MS/MS scan. The frames with MS/MS scan were matched to imported MASCOT results. The ratio of peptides between samples was determined from the variance-weighted average of the ratios in frames, which matched the peptides in the MS/MS spectrum. The ratios of peptides were further integrated to determine the ratio of the corresponding protein. In the differential analysis of protein abundance, total ion current was used for normalization. The requirement for the identification of a protein was a minimum of two matched peptides and two unique peptides. Significant changes in the abundance of proteins between the control and treated samples were analyzed (*p* < 0.05).

### Analysis of protein function and subcellular localization

Protein functions were categorized using MapMan bin code (Usadel et al., 2005). NetNGlyc (http://www.cbs.dtu.dk/services/NetNGlyc/) (Gupta and Brunak, 2002) and N-Glycosite (http://www.hiv.lanl.gov/content/sequence/GLYCOSITE/glycosite.html) (Zhang et al., 2004) were used to predict the N-glycosylation consensus sequence within the protein moiety of identified proteins. The identified proteins were predicted for the presence of N-terminal ER targeting signal peptide with SignalP (http://www.cbs.dtu.dk/services/SignalP/), as this is essential for co-translational translocation of N-glycosylation in the ER (Emanuelsson et al., 2007). Wolf PSORT (http://wolfpsort.org/) and TargetP (http://www.cbs.dtu.dk/services/TargetP/) were used to determine the predicted subcellular locations of the proteins.

### RNA extraction and quantitative reverse transcription polymerase chain reaction analysis

A portion (100 mg) of samples was ground into powder in liquid nitrogen with a sterilized mortar and pestle. Total RNA was extracted from the tissue powder using an RNeasy Plant Mini kit (Qiagen, Valencia, CA, USA). RNA was reverse-transcribed using an iScript cDNA Synthesis kit (Bio-Rad, Hercules, CA, USA) according to the manufacturer's instructions. qRT-PCR was performed in a 10 μL reaction assay using SsoAdvanced SYBR Green Universal Supermix (Bio-Rad) and a MyiQ single-color real-time PCR detection system (Bio-Rad). The PCR conditions were as follows: 95°C for 210 s, then 45 cycles of 95°C for 30 s, 60°C for 30 s, and 72°C for 30 s. Gene expression was normalized using 18S rRNA as an internal control. The primers were designed using the Primer3 web interface (http://frodo.wi.mit.edu). Specificity of primers used for the analysis was checked by the BLASTN search against the Phytozome-*G.max* database with the designed primers sequences as queries (Supplemental Table 1) and by melting curve analysis.

### Statistical analysis

The statistical significance of the results was evaluated with the Student's *t*-test. All calculations were performed by using Graphpad software (version 5.0). A *p* < 0.05 was considered to be statistically significant.

## Results and discussion

### Glycoprotein purification

**Figure 1:**
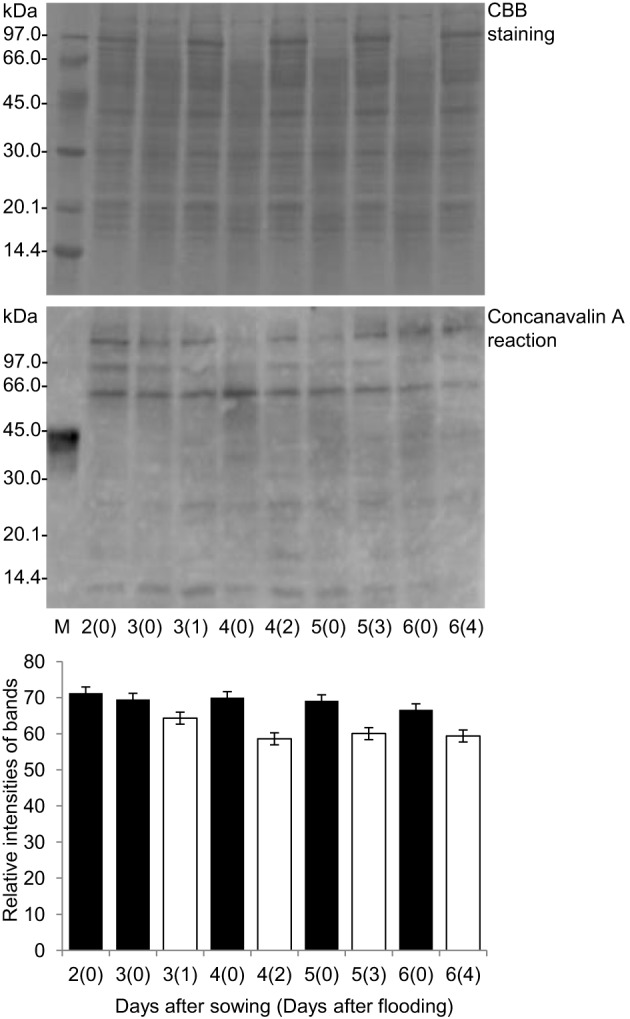
**Effects of flooding stress on glycoproteins stained by ConA**. Two-day-old soybeans were flooded for 1, 2, 3, and 4 days (white columns). Untreated plants served as controls (black columns). Proteins were reacted with ConA antibody. The pattern of CBB staining was used as a loading control. The relative band intensities were calculated using ImageJ software. The analyzed protein samples are shown in Supplemental Figure 1.

To characterize the glycoproteome of soybean roots submitted to a flooding stress, we have in the present work used a protocol previously described in our studies (Komatsu et al., 2013, 2014; Nanjo et al., 2013; Yin et al., 2014). In this protocol, soybean seeds were germinated under optimal control conditions for 2 days and growth is then continued either in control conditions or in flooding stress conditions. Proteomic analysis of the total protein extracts confirm the presence of flooding stress protein markers in the stressed roots, as alcohol dehydrogenase and HSP70 (Komatsu et al., 2013, 2014; Nanjo et al., 2013; Yin et al., 2014). Thereby this protocol was further used to characterize impact of the flooding stress on the glycoproteome of soybean roots. In order to understand the soybean response toward flooding stress, glycoproteomic analysis was performed according to the protocol depicted in Supplemental Figure 1. Proteins extracted from the soybean roots were separated by SDS-PAGE and cross-reacted with ConA antibody (Supplemental Figure 2). The relative band intensities were calculated. The accumulation level of the extracted glycoproteins decreased slightly but significantly under flooding stress as compared to the controls (Figure 1). As the 4-day-old soybeans flooded for 2 days exhibited a significantly decreased accumulation of glycoproteins as compared to controls, this plant sample was further used for glycoproteomic analysis, which was performed using the enriched glycoprotein fraction, as described under materials and methods. The extent of glycoproteins present in the total protein extracts and in the glycoprotein-enriched extracts was compared by Western blotting using ConA antibody (Supplemental Figure 2). It is clear that the intensities of glycoprotein bands were higher in the glycoprotein-enriched protein extracts than in corresponding total protein extracts, testifying the efficiency of the presently used protocol. ConA lectin has broad specificity for high mannose, hybrid, and biantennary complex-type N-glycan (Madera et al., 2008). The present results indicated that glycoproteins were selectively enriched using the ConA lectin resin (Supplemental Figure 2). However, to reduce the false discovery rate for the glycoprotein enrichment, specific glycan-binding lectin could be used. Indeed, in mammals, the combination of lectins enriched different protein subsets (Lee et al., 2010). Therefore, in future work, specific glycan-binding lectin could be used to improve the glycoprotein in soybean roots.

### Identification of enriched glycoproteins using a gel-free proteomics technique

To understand role of the glycoproteins whose accumulation levels changed under flooding stress in soybean roots, glycoproteomics was performed as described in Supplemental Figure 1. In the differential analysis of the glycoproteins, the accumulation levels of 149 proteins were significantly changed in 4-day-old soybean roots compared to the 2-day-old roots under control conditions (Supplemental Table 2). These identified proteins were analyzed for the presence of N-glycosylation sites within the protein moiety using NetNGlyc software, and 111 proteins were found to contain the putative N-glycosylation site. Out of these 111 proteins, the accumulation level of 51 glycoproteins increased and that of 60 glycoproteins were decreased significantly (Supplemental Table 2). SIEVE software was used for the comparison of relative abundance of proteins under control unstressed conditions from three biological replicates (Supplemental Table 3). From this analysis, 87 proteins differentially accumulated following 2 days of flooding stress, with 41 and 46 proteins showing increased and decreased abundance, respectively. From these, 69 proteins contained the putative N-glycosylation site with 34 and 35 proteins exhibiting increased and decreased accumulation levels, respectively (Supplemental Table 4). Peptide sequences and the SIEVE data for three biological replicates were also listed (Supplemental Table 5) highlighting the changes in protein abundance occurring under flooding conditions. Out of the glycoproteins exhibiting changes in accumulation levels under 2 days of flooding stress, concanavalin A like lectin kinase protein (Glyma09g27700.1), polygalacturonase inhibiting protein 1 (Glyma05g25370.1), and SNF1 related protein kinase regulatory subunit gamma 1 (Glyma17g13880.2) accumulated more than 10-fold under flooding stress compared to control conditions, while peroxidase superfamily protein (Glyma12g32160.1), evolutionarily conserved C terminal region (Glyma08g13130.1), and nucleolin (Glyma11g10790.1) showed decreased abundance under the flooding stress conditions.

Among the identified glycoproteins, most of them have already been reported in response to flooding stress in soybean. This was, for example, the case for the polygalacturonase inhibiting proteins, peroxidase, and glyceraldehyde 3 phosphate dehydrogenase (Komatsu et al., 2012; Nanjo et al., 2013). In other species, the homologs of these proteins were shown to display relatively similar behavior under stress conditions. For example, glyceraldehyde 3 phosphate dehydrogenase displayed increased accumulation in *Solanum tuberosum* (Laxalt et al., 1996) and in maize (Chalivendra and Martin, 2003). Also, in rice glycosyl hydrolases were reported to be upregulated under submergence stress conditions (Opassiri et al., 2007). While clathrin heavy chain homologs have been shown to accumulate under salt stress (McLoughlin et al., 2013).

Polygalacturonase inhibiting proteins have been reported to be involved in impairing seed germination by inhibiting pectin degradation, which in turn is regulated by the transcription factor AB15 (Kanai et al., 2010). Under water deficit conditions, abscisic acid accumulation is greater in soybean seedling root tips as compared to other tissues. Komatsu et al. (2013) reported that abscisic acid, through the control of energy conservation via the glycolytic system, enhances the flooding tolerance of soybean root. Ahsan et al. (2005) reported that polygalacturonase is upregulated in response to various abiotic stresses like cold and salinity. In soybean, two polygalacturonase inhibiting protein members have been reported to be upregulated in response to pathogenic infection (Ovidio et al., 2004), one of which was found to show increased accumulation under drought and flooding stress in *Lathyrus sativus* (Tamburino et al., 2012). In agreement with this, Komatsu et al. (2009) reported an increase of these polygalacturonase inhibiting proteins under flooding stress in soybean roots. In the present study, we observed increased accumulation of polygalacturonas inhibiting proteins under flooding stress.

In this study, the accumulation level of glyceraldehyde 3 phosphate dehydrogenase increased under flooding stress (Supplemental Table 4). Previous reports have indicated that under low oxygen conditions, glycolysis and carbohydrate metabolism related proteins are activated, which might be related to the response under energy deprived conditions (Huang and Johnson, 1995). In soybean root, the glyceraldehyde 3 phosphate dehydrogenase protein has been reported to show increased accumulation under flooding stress (Nanjo et al., 2010). Altogether, these results lend further support for the role of the presently characterized glycoproteins in flooding stress response and suggest that plants remodel their glycoproteome to cope with unfavorable environmental conditions.

### Functional analysis and subcellular localization of glycoproteins

**Figure 2:**
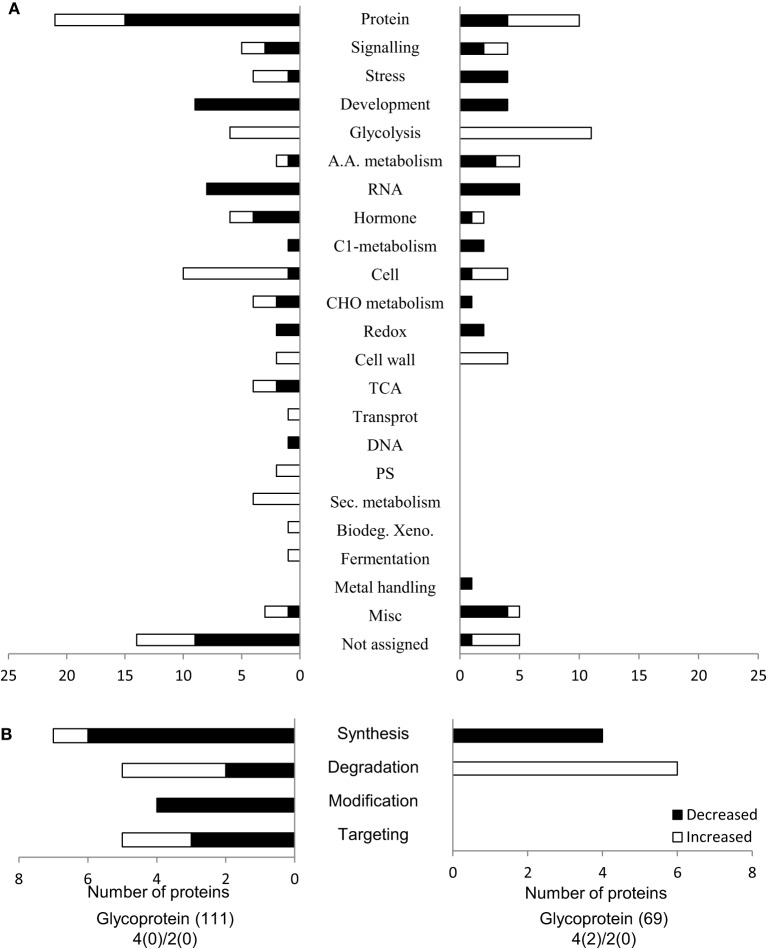
**Functional categorization of the identified glycoproteins. (A)** MapMan bin code was used to predict the functional categorization of the identified glycoproteins. The X-axis indicates the number of identified proteins. Filled bars and open bars indicate increased and decreased glycoproteins in soybean roots under flooding stress, respectively. **(B)** Categorization of the proteins related to protein synthesis, degradation, and post-translational modifications. Abbreviations: Protein, Protein synthesis/degradation/post-translational modification/targeting; A.A. metabolism, amino acid metabolism; RNA, RNA processing/transcription/binding; C1-metabolism, Carbon 1-metabolism; CHO-metabolism, carbohydrate metabolism; DNA, DNA synthesis; PS, photosynthesis; Sec. metabolism, secondary metabolism; Misc., miscellaneous.

To get a better understanding of the biological processes that were altered by flooding stress, the identified glycoproteins were functionally classified. Functional categorization was performed using MapMan bin code (Usadel et al., 2005). Functional categorization showed that the 69 flooding stress responsive glycoproteins are involved in protein synthesis/degradation (19%), glycolysis (15%), cell organization (9%), development (8%), and RNA (7%) (Figure 2A). Proteins related to protein degradation showed significant increased accumulation under flooding stress (Figure 2B). Quantitative proteomics analysis indicated that glycoproteins related to protein degradation, cell wall, and glycolysis display increased abundance under flooding stress, while stress related proteins showed decreased abundance, which is consistent with previous findings (Nanjo et al., 2010, 2011). Among the proteins involved in protein synthesis and degradation, more proteins involved in protein degradation were increased under flooding stress. Under flooding stress, *Rorippa amphibia* shows upregulated genes involved in glycolysis and fermentation that indicate a higher demand for energy production under stress conditions (Akman et al., 2014). In soybean seedlings, an increase in glycolysis related proteins has been reported under flooding stress (Nanjo et al., 2010, 2011). These results suggest that changes in glycolysis related proteins contribute to energy production, and that this could be an acclimation response of soybean roots toward flooding stress.

**Figure 3:**
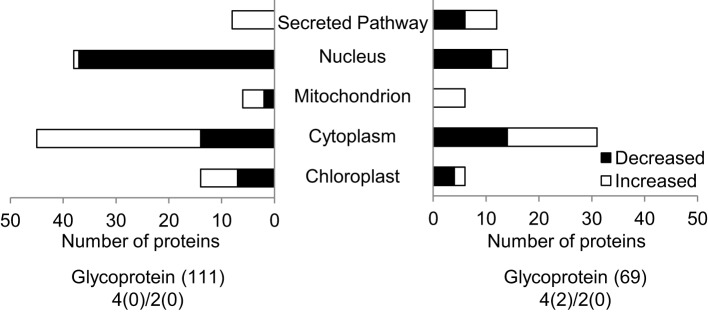
**Subcellular localization of the identified glycoproteins**. Wolf PSORT and TargetP were used to predict the subcellular localization of the identified glycoproteins. The X-axis indicates the number of identified proteins. Filled bars and open bars indicate glycoproteins whose accumulation levels increased and decreased in soybean roots under flooding stress, respectively.

**Figure 4:**
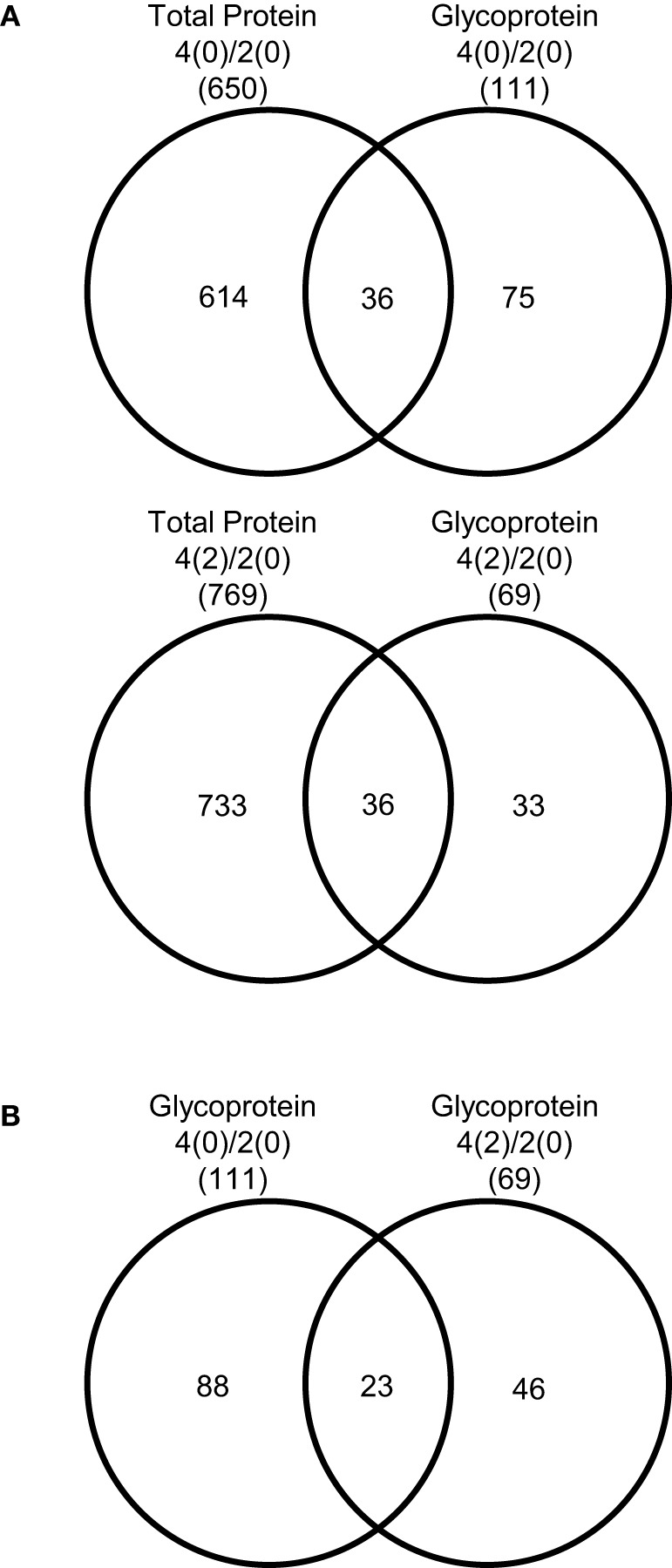
**Comparison of the proteins identified from soybean root. (A)** Total proteins identified from 4-day-old soybean roots compared to the glycoproteins in the control. Total proteins identified from 2-day-old soybean roots flooded for 2 days were compared to the glycoproteins. **(B)** Glycoproteins identified from 4-day-old flooded for 2 days soybean roots compared to glycoproteins identified from 4-days-old soybean roots.

To further decipher the role of the presently identified flooding responsive glycoproteins, a subcellular localization analysis of these identified glycoproteins was performed using Wolf PSORT (Horton et al., 2007) and TargetP (Emanuelsson et al., 2007) (Figure 3). Out of the presently identified 69 glycoproteins, 31 were predicted to be localized in the cytoplasm (45%), while 14 and 12 glycoproteins were localized in the nucleus (20%) and secretory pathway (17%), respectively (Figure 3). Proteins related to the secretory pathway showed significant decreased abundance under flooding stress. Secretory pathway related proteins like peroxidase superfamily proteins and glycosyl hydrolases displayed decreased accumulation levels in soybean roots under flooding stress (Figure 4, Table 1).

**Table 1:** **List of identified glycoproteins of root from 4-day-old and 4-day-old soybean flooded for 2 days compared with 2-day-old soybean**.

**Protein ID**	**Description**	**M.P**.	**Ratio 4(0)**	**Ratio 4(2)**	**Subcellular localization**	**Function**	**N-Glycosylation site**		**Secretory pathway (Signal P software)**
							**NetNGlyc software**	**N-Glycosite software**	
1. Glyma12g09940.2	FAD/NAD(P) binding oxidoreductase family protein	5	14.20	8.17	Cytoplasm	Not assigned	27 NVTL, 76 NITE	NVTL, NITET	N
							27 NVTL, 76 NITE, 201	NVTL, NITE, NKTY	N
2. Glyma11g18320.1	FAD/NAD(P) binding oxidoreductase family protein	9	9.22	8.14	Cytoplase	Not assigned	NKTY		
3. Glyma03g31670.1	RNA binding KH domain containing protein	2	0.67	0.89	Cytoplasm	RNA	23 NHSD, 424 NISY	NHSD, NISY	N
4. Glyma04g07841.1	SHK1 binding protein 1	2	0.61	0.70	Chloroplast	C1-metabolism	251 NHSI, 547 NDTG	NHSI, NDTG	N
							68 NTSL, 146 NHTY, 172	NTS, NHTY, NGTG, ND S,	N
5. Glyma13g42320.1	Lipoxygenase 1	7	0.49	0.69	Peroxisome	Hormone metabolism	NGTG, 489 NDSC, 641 NDSE	NDSE	
6. Glyma01g42840.1	Glutathione peroxidase 6	4	0.40	0.64	Cytoplasm	Redox	48 NYTE, 132 NFSK	NYT, NFS	N
7. Glyma11g02630.1	Glutathione peroxidase 6	6	0.40	0.64	Cytoplasm	Redox	48 NYTE, 132 NFSK	NYT, NFSK	N
							246 NVSD, 340 NFTY,	NVSDA, N FT, N AT,	N
8. Glyma07g32480.1	Apoptosis inhibitory protein 5 (API5)	5	0.53	0.58	Cytoplasm	Development	360 NATN, 416 NKSM, 437 NATT, 469 NLSW, 495 NGSN	NKSMA NATT, NL SW, NGSN	
							247 NVSD, 341 NFTY,	NVSD, NFTY, NATN	N
9. Glyma13g24090.2	Apoptosis inhibitory protein 5 (API5)	5	0.53	0.58	Cytoplasm	Development	361 NATN, 417 NKSM, 438 NATT, 470 NLSW, 496 NGSN	NKSM, NAT, N LS, NGSN	
10.							70 NETS, 106 NSSA, 122	NETS, NSSA, NHSE,	N
Glyma07g13900.1	Hyaluronan	6	0.49	0.52	Nucleus	RNA	NHSE 313 NITE	NITE	
11. Glyma07g38790.1	NAD(P) binding Rossmann fold superfamily protein	5	0.41	0.51	Cytoplasm	Misc	182 NSTS	NSTS	N
12. Glyma09g25830.2	CAP160 protein	11	0.17	0.50	Nucleus	Not assigned	491 NQTT, 917 NQSS	NQTT, NQSS	N
13. Glyma05g04290.1	Glycosyl hydrolases family 32 protein	8	2.80	0.46	Chloroplast	Major CHO metabolism	208 NESV, 221 NPSD, 273 NKTG, 616 NATE	NESV, NKTG, NATE	N
14. Glyma13g39790.1	ABC transporter family protein	2	0.48	0.45	Cytoplasm	Protein.synthesis	228 NPTI, 496 NLSD	NLSD	N
15. Glyma11g12480.1	Cold circadian rhythm and RNA binding 2	3	0.27	0.42	Nucleus	RNA	78 NITV 104 NRSG	NITV, NRSG	N
16. Glyma11g12490.1	Cold circadian rhythm and RNA binding 2	3	0.27	0.42	Cytoplasm	RNA	78 NITV 135 NGSR	NITV, NGSR	N
							24 NASE, 135 NGTT, 278	NASE, NGTT, NLSL,	N
17. Glyma03g27030.1	DNAJ homolog	2	0.46	0.39	Cytoplasm	Stress abiotic	NLSL		
							128 NKTG, 344 NKTA,	NKTG, NKTA,	N
18. Glyma10g07410.1	Embryonic cell protein 63	6	0.35	0.23	Nucleus	Development	367 NVSG		
							79 NSTT, 92 NLTV, 151	NSTT, NLTV, NLTE, NFTT,	Y
19. Glyma12g32160.1	Peroxidase superfamily protein	2	3.17	0.22	Cytoplasm	Stress abiotic	NLTE 165 NFTT, 206 NFTG, 237 NTTK	NFTG, NTTK	
							79 NSTT, 92 NLTV, 165	NSTT, NLTV, NITT, NFTG,	Y
20. Glyma12g32170.1	Peroxidase superfamily protein	2	3.17	0.22	Cytoplasm	Misc	NITT 206 NFTG, 237 NTTK	NTTK	
							79 NSTT, 92 NLTV, 165	NSTT, NLTV, NITT, NFTG,	Y
21. Glyma13g38300.1	Peroxidase superfamily protein	2	3.17	0.22	Cytoplasm	Misc	NITT 206 NFTG, 237 NTTK, 292 NFSA	NTTK, NFSA	
							77 NSTT, 90 NLTV, 149	NSTT, NLTV, NLTE, NFTT,	Y
22. Glyma13g38310.2	Peroxidase superfamily protein	2	3.17	0.22	Cytoplasm	Stress abiotic	NLTE 163 NFTT, 204 NFTG, 235 NTTK	NFTG, NTTK	
							176 NIST, 248 NQSA, 253	NIST, NQSA, NGTL,	N
23. Glyma11g10790.1	Nucleolin like 2	8	0.31	0.03	Nucleus	Protein synthesis	NGTL 562 NSSN, 567 NNSS, 568 NSSQ	NSSN, NNSS	

*Protein ID, according to the Phytozome database; M.P., matched peptide; Ratio, relative abundance of a protein from 4 day old soybean compared to 2-days old soybean root; ND, no description; N-Glycosylation site, Presence of the N-glycosylation site in protein moiety based on the N-Glycosite Software and NetNGlyc Software. Secretory Pathway, signal peptide presence based on the SignalP 4.1*.

Plant peroxidases are secreted glycoproteins involved in numerous mechanisms, like cell elongation, cell wall construction, and defense against pathogens (Kukavica et al., 2012). Peroxidase super family proteins are induced by drought in wild watermelons (Yoshimura et al., 2008) and maize roots (Degenhardt and Gimmler, 2000), suggesting enhanced lignin production. Peroxidases have been reported to be in lower abundance under flooding stress (Shi et al., 2008; Komatsu et al., 2010, 2012). In soybean root, peroxidase genes have been reported to be downregulated under flooding stress (Nishizawa et al., 2013). Under flooding stress, the soybean seedlings show reduced growth (Hashiguchi et al., 2009). This growth inhibition functions to conserve energy under stress conditions and promotes survival by anaerobic respiration (Nanjo et al., 2011). The observed decreased abundance of peroxidase superfamily proteins under flooding stress (Table 1, Supplemental Table 6), suggesting decreased lignin synthesis and ultimately a reduction in cell wall formation. In agreement, a proteomic analysis of soybean root cell wall proteins disclosed the suppression of the lignification process under flooding stress conditions (Komatsu et al., 2010). This could be a plant strategy to conserve energy under stress conditions.

Glycosyl hydrolase proteins are involved in the biosynthesis of glycans and plant defense. Glycosyl hydrolases family 32 proteins have been reported to be of the acid invertase type that functions as cell wall invertases (Lammens et al., 2009). In the cell, glycosyl hydrolases are responsible for the cleavage of glycosidic linkages and play a role in glycan processing (Trincone and Giordano, 2006). These proteins catalyze the hydrolysis of glycosidic bonds between sugars and other moieties (Henrissat and Davies, 1997). In rice, glycosyl hydrolases are induced under stress conditions (Opassiri et al., 2007). In this study, glycosyl hydrolases showed decreased abundance in soybean roots submitted to flooding stress (Table 1). These results suggest that flooding stress might cause a reduction in glycan synthesis that ultimately leads to decreased glycoprotein synthesis in soybean roots.

### Effect of flooding stress on N-glycosylation and ER-related genes

**Figure 5:**
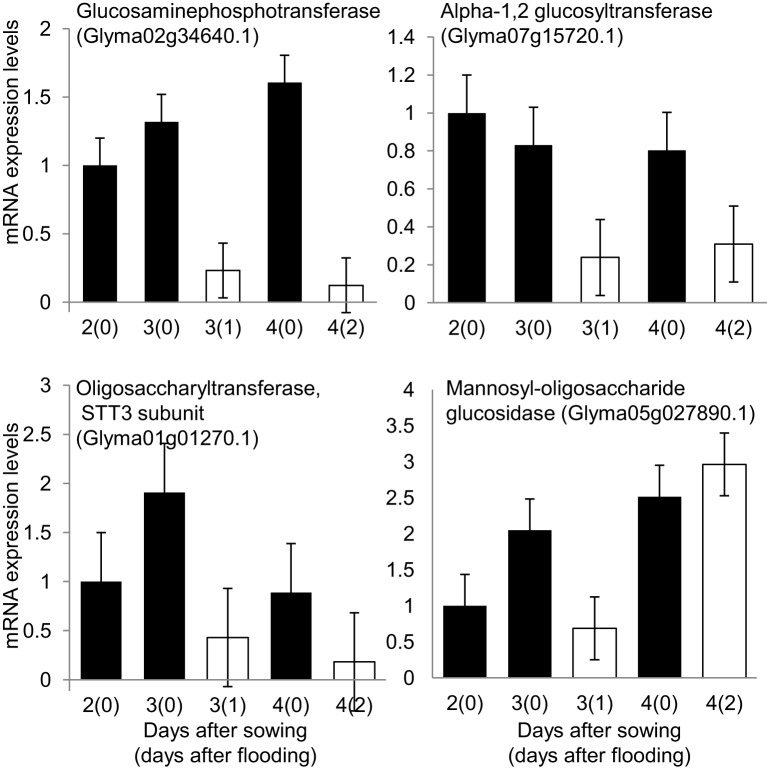
**Effects of flooding stress on mRNA expression levels of N-glycosylation related genes**. Two-day-old soybeans were treated with flooding for 1 and 2 days (white column). Untreated plants served as a control (black column). RNA extracted from roots of soybean was analyzed by qRT-PCR with specific primers for N-glycosylation genes (Supplemental Table 1; Supplemental Figure 1 for the analyzed samples). mRNA expression levels indicate relative mRNA abundance normalized against 18S rRNA abundance. The data shows mean ± SE values from three independent biological replicates. Among N-glycosylation related genes, glucosaminephosphotransferase (Glyma02g34640.1), alpha-1, 2 glucosyltransferase (Glyma07g15720.1), oligosaccharyltransferase, STT3 subunit (Glyma01g01270.1), and mannosyl-oligosaccharide glucosidase (Glyma05g27890.1) were selected.

**Figure 6:**
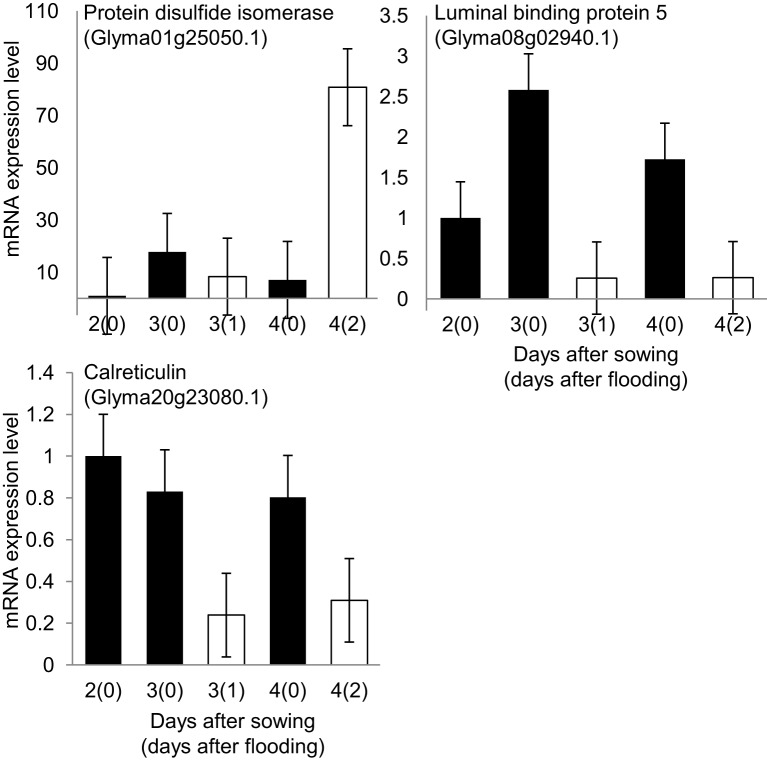
**Effects of flooding stress on mRNA expression levels of ER related genes**. Two-day-old soybeans were treated with flooding for 1 and 2 days (white column). Untreated plants served as controls (black columns). RNA extracted from roots of soybeans was analyzed by qRT-PCR with specific primers for ER protein genes (Supplemental Table 1). mRNA expression levels indicate relative mRNA abundance normalized against 18S rRNA abundance. The data shows mean ± SE values from three independent biological replicates. Among ER related genes, protein disulfide isomerase (Glyma01g25050.1), luminal binding protein 5 (Glyma08g02940.1), and calreticulin (Glyma20g23080.1) were selected.

To understand the effect of flooding stress on the process of N-glycosylation, the mRNA expression levels of four N-glycosylation-related genes (Figure 5) and three ER-related genes (Figure 6) were analyzed using qRT-PCR. Among N-glycosylation-related genes, glucosaminephosphotransferase (Glyma02g34640.1), alpha-1, 2 glucosyltransferase (Glyma07g15720.1), STT3 subunit of oligosaccharyltransferase (Glyma01g01270.1), and mannosyl-oligosaccharide glucosidase (Glyma05g27890.1) were analyzed. Among ER-related genes, protein disulfide isomerase (Glyma01g25050.1), luminal binding protein 5 (Glyma08g02940.1), and calreticulin (Glyma20g23080.1) were analyzed. In these experiments, 2-day-old soybeans were flooded for 1 and 2 days. Total RNA was extracted and analyzed. Untreated plants served as controls.

The mRNA expression levels of glucosaminephosphotransferase, an enzyme that is involved in starting the process of N-glycan synthesis at the cytoplasmic face of the ER (Koizumi et al., 1999), alpha-1, 2 glucosyltransferase, and STT3 subunit of oligosaccharyltransferase were downregulated under 1 day of flooding stress (Figure 5). Mannosyl-oligosaccharide glucosidase, which is involved in N-glycan trimming (Gillmor et al., 2002), was downregulated under flooding stress for 1 day and upregulated under flooding stress for 2 days (Figure 5). These results indicated that the process of N-glycosylation and ultimately glycoprotein synthesis was significantly downregulated under 1-day flooding stress. The mRNA expression level of protein disulfide isomerase, was upregulated under flooding stress conducted for 2 days (Figure 6). Luminal binding protein 5, which interacts with polypeptide folding intermediates (Morris et al., 1997) and calreticulin, which is responsible for the folding of newly synthesized polypeptide chains and glycoproteins (Michalak et al., 2002), were downregulated under 1 day of flooding stress. Protein disulfide isomerase inserts disulfides into proteins and provides mechanism to correct errors in disulfide pairing (Gilbert, 1997). In pea roots, protein disulfide isomerase was detected in relatively high abundance under stress conditions. The observed downregulation of luminal binding protein and calreticulin under flooding stress indicates that the protein folding process in the ER had been disrupted because these are important for protein folding. These results indicated that protein folding was disrupted in the ER.

Alpha-1, 2 glucosyltransferase, which is involved in the process of glycan extension (Farid et al., 2011), was downregulated under flooding stress. Farid et al. (2011) reported that alpha-1, 2 glucosyltransferase in *Arabidopsis* is required for lipid-linked oligosaccharide biosynthesis and the abiotic stress response. Furthermore, inactivation of alpha-1, 2 glucosyltransferase results in the activation of the unfolded protein response and increased sensitivity to salt stress. Burda and Aebi (1998) reported that mutants lacking this gene experience decreased glycosylation in *Saccharomyces cerevisiae*. These results suggest that downregulation of the mRNA expression level of alpha-1, 2 glucosyltransferase in soybean root under flooding stress leads to decreased glycosylation.

The mRNA expression level of the STT3 subunit of oligosaccharyltransferase, which is involved in glycan transfer to asparagine residues in target proteins (Koiwa et al., 2003), was downregulated in soybean root under flooding stress (Figure 5). The oligosaccharyltransferase complex governs the central step of N-glycosylation, which transfers the preassembled oligosaccharide to the protein in the ER (Mohorko et al., 2011). In *Arabidopsis*, the STT3 subunit has been reported to control the plant response toward salt/osmotic stress (Koiwa et al., 2003). STT3 subunit deficiency results in protein underglycosylation defects that disturb the biogenesis of heavily glycosylated proteins and ultimately plant innate immunity (Nekrasov et al., 2009). In this way, plants could modify their system to cope with stress conditions. The downregulation of the STT3 subunit of oligosaccharyltransferase might an imporatant part of the response of plant toward flooding stress. Soybean plants under flooding stress might experience underglycosylation that leads to the decreased glycoprotein levels. The downregulation of the genes involved in protein glycosylation was clearly noticed at the proteomic level (Figure 5).

The gene encoding protein disulfide isomerase, which is involved in the formation of disulfide bonds in nascent polypeptide chains (Freedman et al., 1994), was upregulated in soybean roots under flooding stress (Figure 6). Protein disulfide isomerase family proteins are involved in polypeptide folding and the formation of the disulfide bonds in the ER (Freedman et al., 1994). Protein disulfide isomerase is sorted to the ER to behave as a chaperone for reconstructing misfolded proteins in this compartment (Wang et al., 2012), and is involved in the quality control of storage proteins (Kamauchi et al., 2008). Stressful conditions result in the accumulation of misfolded proteins in the ER (Liu and Howell, 2010). These results suggest that under flooding stress conditions, the upregulation of protein disulfide isomerase might help to reduce misfolded proteins in the ER. This upregulation could be a remodeling strategy to reconstruct misfolded proteins and specifically glycoproteins in stressed plants.

## Concluding remarks

Gel-free glycoproteomics proved useful to uncover the mechanisms that are involved in early stages of soybean response against flooding stress. A total of 69 glycoproteins from soybean roots were found to display significant changes in abundance under 2 days of flooding stress. Functional categorization of these identified glycoproteins indicated that the majority were related to glycolysis and protein degradation. Subcellular prediction of these identified glycoproteins indicated their localization to the cytoplasm, nucleus, and secretory pathways. Proteins involved in energy metabolism such as glyceraldehydes 3 phosphate dehydrogenase were found to have increased accumulation under flooding stress. mRNA expression levels of genes involved in the N-glycosylation pathway indicated a downregulation at every step of that pathway. From the present results, and as previously observed (Baerenfaller et al., 2008; Piques et al., 2009; Schwanhäusser et al., 2011; Galland et al., 2014), it appears that mRNA and protein accumulation profiles differ. We suggest that this behavior originates from the various check points in gene expression and also from different extents of protein and mRNA stability levels. Altogether, our present results document for the first time that a flooding stress entails a large modification in the glycoproteome of soybean roots, which may provide new avenues for the selection of novel soybean varieties more resistant to this stress compromising crop yield.

## Accession code

The mass spectrometry proteomics data have been deposited to the ProteomeXchange Consortium (http://proteomecentral.proteomexchange.org) via the PRIDE partner repository (Vizcaíno et al., 2013) with the data set identifier and doi PXD001350.

### Conflict of interest statement

The authors declare that the research was conducted in the absence of any commercial or financial relationships that could be construed as a potential conflict of interest.

## Abbreviations

CBB: Coomassie brilliant blue
ConA: concanavalin A
LC: liquid chromatography
MS: mass spectrometry
ER: endoplasmic reticulum
qRT-PCR: quantitative reverse transcription polymerase chain reaction.
