# Supplementary material for: Quantitative proteomics reveals the effect of protein glycosylation in soybean root under flooding stress
Source: Front Plant Sci. 2014 Nov 18;5:627. doi: 10.3389/fpls.2014.00627 (PMC4235293; doi:10.3389/fpls.2014.00627)
Supplement: Supplementary file 1 [file DataSheet1.ZIP › Supplemental Table 1.docx]

Supplemental Table 1. Primer sequences of Genes selected for qRT-PCR.

| Protein ID^a)^ | Description | Sequence of primers |
| --- | --- | --- |
| Glyma02g34640.1 | UDP-n-acetylglucosamine-dolichyl-phosphate n-acetylglucosaminephosphotransferase | F: 5'-GCTGGGATGACTATGGCTGT-3'  R: 5'-GTCAAACCTTGGCAGTCGAT-3' |
| Glyma07g15720.1 | Alpha-1,2 glucosyltransferase | F: 5'-GGATACCGTTGAGCACTCGT-3' |
|  |  | R: 5'-TCTGTGCAAAATGTGGGGTA-3' |
| Glyma01g01270.1 | Oligosaccharyltransferase, STT3 subunit | F: 5'-TAGTGTATGGGCAGCAGCAG-3' |
|  |  | R: 5'-TTAGCCATGGCAGTTGTCTG-3' |
| Glyma05g27890.1 | Mannosyl-oligosaccharide glucosidase | F: 5'-ACTCTGAGCTTGAGGGTCCA-3' |
|  |  | R: 5'-AGCCGGTAAATGGATGTGAC-3' |
| Glyma01g25050.1 | Protein disulfide isomerase | F: 5'-TCTTGCCGAGAATTTCAGGT-3' |
|  |  | R: 5'-CCAGAGTTGCCATCCTGTTT-3' |
| Glyma08g02940.1 | Luminal binding protein 5 | F: 5'-TGCAGTGAAAGAAGCATTGG-3' |
|  |  | R: 5'-AGCGCAGGGGAATTCTTAAT-3' |
| Glyma20g23080.1 | Calreticulin | F: 5'-GCCAAGAAGCCTGAAGATTG-3' |
|  |  | R: 5'-TTTCACCTGCCACAATTCAA-3' |
| 18S rRNA | X02623.1^b)^ | F: 5'-TGATTAACAGGGACAGTCGG-3' |
|  |  | R: 5'-ACGGTATCTGATCGTCTTCG-3' |

a, Protein ID according to the Phytozome database; b, according to Genebank.
