# Supplementary material for: Quantitative proteomics reveals the effect of protein glycosylation in soybean root under flooding stress
Source: Front Plant Sci. 2014 Nov 18;5:627. doi: 10.3389/fpls.2014.00627 (PMC4235293; doi:10.3389/fpls.2014.00627)
Supplement: Supplementary file 1 [file DataSheet1.ZIP › Supplemental Table 2.docx]

| Supplemental Table 2. List of identified glycoproteins of root from 4-day-old compared to 2-day-old soybean. | | | | | | |  |
| --- | --- | --- | --- | --- | --- | --- | --- |
| Protein ID | Description | M.P. | Ratio | *p-*value | Subcellular localization | Function | Secretory Pathway  (SignalP software) |
| Increased |  |  |  |  |  |  |  |
| 1. Glyma12g09940.2 | FAD/NAD(P) binding oxidoreductase | 5 | 14.2 | 0.03 | Cytoplasm | Not assigned | N |
| 1. Glyma11g18320.1 | FAD/NAD(P) binding oxidoreductase | 9 | 9.22 | 0 | Mitochondrion | Not assigned | N |
| 1. Glyma07g04500.4 | Subtilase family protein | 9 | 7.94 | 0 | Cytoplasm | Protein.degradation | N |
| 1. Glyma16g01090.1 | Subtilase family protein | 10 | 7.43 | 0.01 | Secreted pathway | Protein.degradation | Y |
| 1. Glyma04g16350.1 | prohibitin 4 | 3 | 5.83 | 0.05 | Mitochondrion | Cell | N |
| 1. Glyma18g45260.2 | NAD(P) binding Rossmann fold | 2 | 5.47 | 0.01 | Mitochondrion | Secondary metabolism | N |
| 1. Glyma09g40580.1 | NAD(P) binding Rossmann fold | 3 | 5.01 | 0.03 | Cytoplasm | Secondary metabolism | N |
| 1. Glyma05g35970.1 | profilin 2 | 2 | 4.54 | 0.05 | Cytoplasm | Cell | N |
| 1. Glyma07g15090.1 | profilin 4 | 2 | 4.54 | 0.05 | Cytoplasm | Cell | N |
| 1. Glyma08g03650.1 | profilin 2 | 2 | 4.54 | 0.05 | Cytoplasm | Cell | N |
| 1. Glyma08g03660.1 | profilin 5 | 2 | 4.54 | 0.05 | Cytoplasm | Cell | N |
| 1. Glyma01g03260.1 | alanine 2 oxoglutarate aminotransferase 2 | 2 | 3.87 | 0.04 | Cytoplasm | PS | N |
| 1. Glyma02g04320.4 | alanine 2 oxoglutarate aminotransferase 2 | 2 | 3.87 | 0.04 | Cytoplasm | PS | N |
| 1. Glyma09g40590.1 | NAD(P) binding Rossmann fold | 3 | 3.69 | 0.03 | Cytoplasm | Secondary metabolism | N |
| 1. Glyma08g04370.1 | aldehyde dehydrogenase 2C4 | 5 | 3.38 | 0.05 | Cytoplasm | Fermentation | N |
| 1. Glyma12g32160.1 | Peroxidase superfamily protein | 2 | 3.17 | 0.01 | Secreted pathway | Stress | Y |
| 1. Glyma12g32170.1 | Peroxidase superfamily protein | 2 | 3.17 | 0.01 | Secreted pathway | Misc | Y |
| 1. Glyma13g38300.1 | Peroxidase superfamily protein | 2 | 3.17 | 0.01 | Secreted pathway | Misc | Y |
| 1. Glyma13g38310.2 | Peroxidase superfamily protein | 2 | 3.17 | 0.01 | Secreted pathway | Stress | Y |
| 1. Glyma17g14750.1 | Glycosyl hydrolases family 32 protein | 10 | 3.12 | 0 | Secreted pathway | Major CHO metabolism | N |
| 1. Glyma12g35820.1 | general regulatory factor 9 | 2 | 3.08 | 0.04 | Cytoplasm | Signalling | N |
| 1. Glyma01g37820.1 | NmrA like negative transcriptional regulator | 2 | 2.99 | 0.04 | Cytoplasm | Secondary metabolism | N |
| 1. Glyma05g04290.1 | Glycosyl hydrolases family 32 protein | 8 | 2.8 | 0 | Secreted pathway | Major CHO metabolism | N |
| 1. Glyma11g36620.1 | S adenosyl L homocysteine hydrolase | 3 | 2.78 | 0.03 | Cytoplasm | Amino acid metabolism | N |
| 1. Glyma05g21260.1 | lipoxygenase 1 | 2 | 2.62 | 0.04 | Cytoplasm | Hormone metabolism | N |
| 1. Glyma13g41370.1 | translocon | 4 | 2.59 | 0.04 | Chloroplast | Protein.targeting | N |
| 1. Glyma15g04030.1 | translocon | 4 | 2.59 | 0.04 | Chloroplast | Protein.targeting | N |
| 1. Glyma13g00751.1 | villin 4 | 4 | 2.53 | 0.02 | Cytoplasm | Cell | N |
| 1. Glyma02g38730.1 | Aldolase superfamily protein | 11 | 2.38 | 0.03 | Mitochondrion | Glycolysis | N |
| 1. Glyma15g11530.1 | glyoxalase I homolog | 2 | 2.37 | 0.04 | Cytoplasm | Biodegradation of Xenobiotics | N |
| 1. Glyma09g06390.3 | villin 4 | 5 | 2.2 | 0.02 | Cytoplasm | Cell | N |
| 1. Glyma15g17640.2 | villin 4 | 5 | 2.2 | 0.02 | Cytoplasm | Cell | N |
| 1. Glyma08g24720.1 | MLP like protein 43 | 6 | 2.18 | 0.02 | Cytoplasm | Stress | N |
| 1. Glyma13g42330.1 | lipoxygenase 1 | 10 | 2.17 | 0.04 | Chloroplast | Hormone metabolism | N |
| 1. Glyma18g12900.1 | villin 4 | 2 | 2.16 | 0.03 | Cytoplasm | Cell | N |
| 1. Glyma09g18920.1 | ND | 2 | 2.16 | 0.02 | Cytoplasm | Not assigned | N |
| 1. Glyma14g08240.1 | ND | 2 | 2.16 | 0.02 | Cytoplasm | Not assigned | N |
| 1. Glyma04g04400.2 | Granulin repeat cysteine protease | 3 | 2.08 | 0.04 | Secreted pathway | Protein.degradation | Y |
| 1. Glyma16g07750.1 | ND | 26 | 1.99 | 0 | Nucleus | Not assigned | N |
| 1. Glyma02g00810.1 | Lactate/malate dehydrogenase family protein | 3 | 1.92 | 0.01 | Chloroplast | TCA | N |
| 1. Glyma01g07930.1 | reversibly glycosylated polypeptide 3 | 6 | 1.91 | 0.01 | Cytoplasm | Cell wall | N |
| 1. Glyma02g13330.1 | reversibly glycosylated polypeptide 3 | 6 | 1.85 | 0.03 | Cytoplasm | Cell wall | N |
| 1. Glyma18g48280.4 | ATPase V1 complex subunit B protein | 5 | 1.72 | 0.03 | Cytoplasm | Transport | N |
| 1. Glyma10g00920.1 | Lactate/malate dehydrogenase family protein | 4 | 1.52 | 0 | Chloroplast | TCA | N |
| 1. Glyma07g01540.1 | Ribosomal L28e protein family | 2 | 1.42 | 0.03 | Cytoplasm | Protein | N |
| 1. Glyma06g09320.5 | phosphofructokinase 3 | 4 | 1.37 | 0.03 | Cytoplasm | Glycolysis | N |
| 1. Glyma01g00870.1 | phosphofructokinase 3 | 2 | 1.37 | 0.01 | Chloroplast | Glycolysis | N |
| 1. Glyma07g15170.5 | phosphofructokinase 3 | 2 | 1.37 | 0.01 | Chloroplast | Glycolysis | N |
| 1. Glyma08g03570.1 | phosphofructokinase 3 | 2 | 1.37 | 0.01 | Cytoplasm | Glycolysis | N |
| 1. Glyma05g36050.2 | phosphofructokinase 3 | 3 | 1.36 | 0.05 | Cytoplasm | Glycolysis | N |
| 1. Glyma02g16611.1 | RAN GTPase activating protein 1 | 8 | 1.36 | 0.04 | Cytoplasm | Signalling | N |
| Decreased |  |  |  |  |  |  |  |
| 1. Glyma02g01930.1 | Fumarase 2 | 3 | 0.91 | 0.05 | Cytoplasm | TCA | N |
| 1. Glyma10g02040.4 | Fumarase 1 | 3 | 0.91 | 0.05 | Mitochondrion | TCA | N |
| 1. Glyma01g41790.1 | RGPR related | 3 | 0.83 | 0.04 | Nucleus | Not assigned | N |
| 1. Glyma20g26280.2 | PTEN 2 | 6 | 0.71 | 0.02 | Nucleus | Protein | N |
| 1. Glyma03g31670.1 | RNA binding KH domain containing protein | 2 | 0.67 | 0 | Nucleus | RNA | N |
| 1. Glyma07g00873.1 | lipoxygenase 1 | 2 | 0.67 | 0.05 | Cytoplasm | Hormone metabolism | N |
| 1. Glyma07g03910.1 | lipoxygenase 1 | 2 | 0.67 | 0.05 | Chloroplast | Hormone metabolism | N |
| 1. Glyma19g34470.1 | RNA binding KH domain containing protein | 6 | 0.66 | 0.01 | Nucleus | RNA | N |
| 1. Glyma04g07841.1 | SHK1 binding protein 1 | 2 | 0.61 | 0.03 | Nucleus | C1-metabolism | N |
| 1. Glyma01g28520.1 | Carbohydrate binding like fold | 3 | 0.6 | 0.01 | Mitochondrion | Major CHO metabolism | N |
| 1. Glyma17g14280.1 | HSP70 interacting protein 1 | 9 | 0.59 | 0.02 | Nucleus | Not assigned | N |
| 1. Glyma18g49189.1 | Hyaluronan | 3 | 0.59 | 0.03 | Nucleus | RNA | N |
| 1. Glyma05g33930.1 | eukaryotic translation initiation factor 3C | 10 | 0.57 | 0.03 | Nucleus | Protein | N |
| 1. Glyma07g32480.1 | Apoptosis inhibitory protein 5 (API5) | 5 | 0.53 | 0.01 | Cytoplasm | Development | N |
| 1. Glyma13g24090.2 | Apoptosis inhibitory protein 5 (API5) | 5 | 0.53 | 0.01 | Cytoplasm | Development | N |
| 1. Glyma19g32540.2 | nucleolar protein gar2 related | 3 | 0.52 | 0.02 | Nucleus | DNA | N |
| 1. Glyma12g09390.1 | NAD ADP ribosyltransferases | 10 | 0.51 | 0.02 | Nucleus | Protein | N |
| 1. Glyma08g10671.1 | SRP72 RNA binding domain | 6 | 0.49 | 0.02 | Nucleus | Protein.targeting | N |
| 1. Glyma13g42320.1 | lipoxygenase 1 | 7 | 0.49 | 0 | Chloroplast | Hormone metabolism | N |
| 1. Glyma07g13900.1 | Hyaluronan | 6 | 0.49 | 0 | Nucleus | RNA | N |
| 1. Glyma13g39790.1 | ABC transporter family protein | 2 | 0.48 | 0.04 | Cytoplasm | Protein.synthesis | N |
| 1. Glyma11g19070.2 | NAD ADP ribosyltransferases | 14 | 0.48 | 0.05 | Nucleus | Protein.postranslational modification | N |
| 1. Glyma08g09270.3 | ubiquitin protein ligase 1 | 3 | 0.48 | 0.03 | Nucleus | Protein.degradation | N |
| 1. Glyma05g27690.1 | SRP72 RNA binding domain | 8 | 0.47 | 0 | Nucleus | Protein.targeting | N |
| 1. Glyma08g12140.1 | tubulin alpha 5 | 10 | 0.46 | 0 | Cytoplasm | Cell | N |
| 1. Glyma03g27030.1 | DNAJ homologue 2 | 2 | 0.46 | 0 | Nucleus | Stress | N |
| 1. Glyma19g43790.1 | Calcium binding EF hand family protein | 6 | 0.43 | 0 | Nucleus | Signalling.calcium | N |
| 1. Glyma16g03250.1 | Nucleic acid binding OB fold like protein | 2 | 0.43 | 0 | Cytoplasm | Protein.synthesis | N |
| 1. Glyma19g44160.2 | Nucleic acid binding OB fold like protein | 2 | 0.43 | 0 | Cytoplasm | Protein.synthesis | N |
| 1. Glyma03g41180.1 | Calcium binding EF hand family protein | 6 | 0.42 | 0 | Nucleus | Signalling.calcium | N |
| 1. Glyma10g30930.2 | Calcium binding EF hand family protein | 5 | 0.42 | 0 | Nucleus | Signalling.calcium | N |
| 1. Glyma07g26980.2 | UDP Glycosyltransferase | 2 | 0.42 | 0 | Cytoplasm | Minor CHO metabolism | N |
| 1. Glyma20g36530.1 | Calcium binding EF hand family protein | 6 | 0.42 | 0 | Nucleus | Protein.postranslational modification | N |
| 1. Glyma02g43770.1 | serine/threonine protein phosphatase | 3 | 0.42 | 0.04 | Nucleus | Protein.degradation | N |
| 1. Glyma19g35151.1 | Nuclear transport factor 2 (NTF2) | 4 | 0.41 | 0.01 | Nucleus | Protein.targeting | N |
| 1. Glyma07g38790.1 | NAD(P) binding Rossmann fold | 5 | 0.41 | 0.01 | Nucleus | Misc | N |
| 1. Glyma01g04820.1 | glycine rich protein | 5 | 0.4 | 0.01 | Nucleus | Not assigned | N |
| 1. Glyma01g42840.1 | glutathione peroxidase 6 | 4 | 0.4 | 0 | Cytoplasm | Redox | N |
| 1. Glyma11g02630.1 | glutathione peroxidase 6 | 6 | 0.4 | 0 | Cytoplasm | Redox | N |
| 1. Glyma15g39924.1 | glycine rich protein | 2 | 0.39 | 0.04 | Cytoplasm | Not assigned | N |
| 1. Glyma02g02690.1 | glycine rich protein | 8 | 0.37 | 0.01 | Nucleus | Not assigned | N |
| 1. Glyma14g39850.3 | Ubiquitin like superfamily protein | 3 | 0.36 | 0.03 | Nucleus | Protein.synthesis | N |
| 1. Glyma10g07410.1 | embryonic cell protein 63 | 6 | 0.35 | 0.02 | Nucleus | Development | N |
| 1. Glyma08g10850.2 | urease | 7 | 0.34 | 0.01 | Cytoplasm | Amino acid metabolism | N |
| 1. Glyma11g10790.1 | nucleolin like 2 | 8 | 0.31 | 0.02 | Nucleus | Protein.synthesis | N |
| 1. Glyma20g28460.1 | cupin family protein | 8 | 0.29 | 0.02 | Chloroplast | Development | Y |
| 1. Glyma18g05805.1 | DEAbox RNA helicase family protein | 3 | 0.28 | 0.05 | Chloroplast | Hormone metabolism | N |
| 1. Glyma11g12480.1 | cold circadian rhythm and rna binding 2 | 3 | 0.27 | 0.02 | Chloroplast | RNA | N |
| 1. Glyma11g12490.1 | cold circadian rhythm and rna binding 2 | 3 | 0.27 | 0.02 | Chloroplast | RNA | N |
| 1. Glyma18g06560.1 | TBP associated factor 15B | 4 | 0.24 | 0.04 | Nucleus | RNA | N |
| 1. Glyma20g28640.1 | cupin family protein | 7 | 0.23 | 0.01 | Chloroplast | Development | Y |
| 1. Glyma20g28660.1 | cupin family protein | 10 | 0.19 | 0.01 | Nucleus | Development | Y |
| 1. Glyma20g28650.2 | cupin family protein | 9 | 0.18 | 0.01 | Nucleus | Development | Y |
| 1. Glyma09g25830.2 | CAP160 protein | 11 | 0.17 | 0.05 | Nucleus | Not assigned | N |
| 1. Glyma10g39170.1 | cupin family protein | 6 | 0.17 | 0.03 | Nucleus | Development | Y |
| 1. Glyma05g08030.1 | ND | 2 | 0.13 | 0.04 | Nucleus | Not assigned | N |
| 1. Glyma09g31740.2 | ND | 2 | 0.11 | 0.01 | Cytoplasm | Not assigned | N |
| 1. Glyma10g39150.1 | cupin family protein | 13 | 0.09 | 0 | Nucleus | Development | Y |
| 1. Glyma08g44150.1 | RNA binding (RRM/RBD/RNP motifs) | 2 | 0.08 | 0.02 | Nucleus | RNA | N |
| 1. Glyma16g31331.1 | CAP160 protein | 4 | 0.07 | 0.02 | Nucleus | Not assigned | N |

Protein ID, according to the Phytozome database; M.P., matched peptide; Ratio, relative abundance of a protein from 4 day old soybean compared to 2-days old soybean root; ND, no description; Secretory Pathway, signal peptide presence based on the SignalP 4.1 Server.
