# Supplementary material for: Quantitative proteomics reveals the effect of protein glycosylation in soybean root under flooding stress
Source: Front Plant Sci. 2014 Nov 18;5:627. doi: 10.3389/fpls.2014.00627 (PMC4235293; doi:10.3389/fpls.2014.00627)
Supplement: Supplementary file 1 [file DataSheet1.ZIP › Supplemental Table 4.docx]

| Supplemental Table 4. List of identified glycoproteins of root from 4-day-old flooded for 2 days compared to 2-day-old soybean. | | | | | | | |
| --- | --- | --- | --- | --- | --- | --- | --- |
| Protein ID | Description | M.P. | Ratio | *p-*value | Subcellular Localization | Function | Secretory Pathway  SignalP software |
| Increased |  |  |  |  |  |  |  |
| 1. Glyma09g27700.1 | Concanavalin A like lectin protein kinase family protein | 3 | 136.7 | 0.02 | Secreted pathway | Signalling | N |
| 1. Glyma05g25370.1 | polygalacturonase inhibiting protein 1 | 3 | 86.98 | 0.04 | Secreted pathway | Cell wall | Y |
| 1. Glyma17g13880.2 | SNF1 related protein kinase regulatory subunit gamma 1 | 5 | 16.74 | 0.00 | Cytoplasm | Cell wall | N |
| 1. Glyma05g03300.1 | SNF1 related protein kinase | 4 | 14.38 | 0.00 | Cytoplasm | Cell wall | N |
| 1. Glyma20g35240.1 | Eukaryotic aspartyl protease family protein | 3 | 9.01 | 0.01 | Secreted pathway | Protein | Y |
| 1. Glyma10g32380.1 | Eukaryotic aspartyl protease family protein | 3 | 8.63 | 0.03 | Secreted pathway | Protein | Y |
| 1. Glyma12g09940.2 | FAD/NAD(P) binding oxidoreductase family protein | 5 | 8.17 | 0.05 | Cytoplasm | Not assigned | N |
| 1. Glyma11g18320.1 | FAD/NAD(P) binding oxidoreductase family protein | 9 | 8.14 | 0.00 | Mitochondrion | Not assigned | N |
| 1. Glyma18g01330.1 | glyceraldehyde 3 phosphate dehydrogenase C subunit 1 | 8 | 6.13 | 0.00 | Cytoplasm | Glycolysis | N |
| 1. Glyma11g37360.1 | glyceraldehyde 3 phosphate dehydrogenase C subunit 1 | 9 | 5.82 | 0.00 | Cytoplasm | Glycolysis | N |
| 1. Glyma05g06420.1 | glyceraldehyde 3 phosphate dehydrogenase C2 | 5 | 5.68 | 0.00 | Mitochondrion | Glycolysis | N |
| 1. Glyma19g22780.1 | glyceraldehyde 3 phosphate dehydrogenase C2 | 5 | 5.68 | 0.00 | Mitochondrion | Glycolysis | N |
| 1. Glyma06g09770.1 | Rhamnogalacturonate lyase family protein | 7 | 5.26 | 0.03 | Cytoplasm | Cell wall | N |
| 1. Glyma13g34520.1 | D mannose binding lectin protein | 4 | 4.85 | 0.02 | Secreted pathway | Misc | Y |
| 1. Glyma06g18120.1 | glyceraldehyde 3 phosphate dehydrogenase C subunit 1 | 5 | 4.01 | 0.00 | Mitochondrion | Glycolysis | N |
| 1. Glyma04g36870.3 | glyceraldehyde 3 phosphate dehydrogenase C subunit 1 | 6 | 3.81 | 0.00 | Cytoplasm | Glycolysis | N |
| 1. Glyma04g36860.1 | glyceraldehyde 3 phosphate dehydrogenase C2 | 5 | 3.79 | 0.00 | Mitochondrion | Glycolysis | N |
| 1. Glyma06g18110.1 | glyceraldehyde 3 phosphate dehydrogenase C2 | 5 | 3.74 | 0.00 | Mitochondrion | Glycolysis | N |
| 1. Glyma05g36310.1 | ACC oxidase 1 | 2 | 3.5 | 0.00 | Cytoplasm | Hormone metabolism | N |
| 1. Glyma01g38580.1 | Clathrin heavy chain | 2 | 3.17 | 0.02 | Cytoplasm | Cell | N |
| 1. Glyma02g39360.1 | Clathrin heavy chain | 2 | 3.17 | 0.02 | Cytoplasm | Cell | N |
| 1. Glyma11g06720.1 | Clathrin heavy chain | 2 | 3.17 | 0.02 | Cytoplasm | Cell | N |
| 1. Glyma18g05071.1 | UDP glucose pyrophosphorylase 2 | 2 | 3.02 | 0.02 | Cytoplasm | Glycolysis | N |
| 1. Glyma11g10430.1 | Translocon associated protein (TRAP) | 2 | 2.88 | 0.01 | Secreted pathway | Not assigned | Y |
| 1. Glyma11g33160.1 | UDP glucose pyrophosphorylase 2 | 3 | 2.42 | 0.02 | Cytoplasm | Glycolysis | N |
| 1. Glyma14g39140.1 | UDP glucose pyrophosphorylase 2 | 9 | 2.41 | 0.03 | Cytoplasm | Glycolysis | N |
| 1. Glyma07g18500.1 | metallopeptidase M24 family protein | 7 | 2.32 | 0.02 | Nucleus | Protein.degradation | N |
| 1. Glyma18g43390.1 | metallopeptidase M24 family protein | 7 | 2.32 | 0.02 | Nucleus | Protein.degradation | N |
| 1. Glyma10g44200.1 | 20S proteasome beta subunit D1 | 5 | 2.02 | 0.01 | Cytoplasm | Protein.degradation | N |
| 1. Glyma20g38971.1 | 20S proteasome beta subunit D1 | 5 | 2.02 | 0.01 | Cytoplasm | Protein.degradation | N |
| 1. Glyma07g05130.1 | alanine aminotransferase 2 | 7 | 1.86 | 0.03 | Cytoplasm | Amino acid metabolism | N |
| 1. Glyma16g01630.4 | alanine aminotransferase 2 | 5 | 1.77 | 0.04 | Chloroplast | Amino acid metabolism | N |
| 1. Glyma03g42580.2 | Protein of unknown function (DUF544) | 2 | 1.69 | 0.03 | Nucleus | Not assigned | N |
| 1. Glyma11g35300.2 | Phosphoinositide specific phospholipase C | 4 | 1.23 | 0.02 | Chloroplast | Signalling | N |
| Decreased |  |  |  |  |  |  |  |
| 1. Glyma03g31670.1 | RNA binding KH domain containing protein | 2 | 0.89 | 0.03 | Nucleus | RNA | N |
| 1. Glyma16g27900.3 | Peroxidase superfamily protein | 2 | 0.74 | 0.05 | Secreted pathway | Misc | Y |
| 1. Glyma12g08430.2 | ABC transporter family protein | 4 | 0.71 | 0.03 | Cytoplasm | Protein.synthesis | N |
| 1. Glyma04g07841.1 | SHK1 binding protein 1 | 2 | 0.7 | 0.02 | Nucleus | C1-metabolism | N |
| 1. Glyma13g42320.1 | lipoxygenase 1 | 7 | 0.69 | 0.02 | Chloroplast | Protein.synthesis | N |
| 1. Glyma18g03290.2 | Ribosomal protein L10 family protein | 3 | 0.69 | 0.04 | Cytoplasm | Hormone metabolism | N |
| 1. Glyma11g02630.1 | glutathione peroxidase 6 | 6 | 0.64 | 0.00 | Cytoplasm | Redox | N |
| 1. Glyma01g42840.1 | glutathione peroxidase 6 | 4 | 0.64 | 0.00 | Cytoplasm | Redox | N |
| 1. Glyma07g32480.1 | Apoptosis inhibitory protein 5 (API5) | 5 | 0.58 | 0.05 | Cytoplasm | Development | N |
| 1. Glyma13g24090.2 | Apoptosis inhibitory protein 5 (API5) | 5 | 0.58 | 0.05 | Cytoplasm | Development | N |
| 1. Glyma15g30110.1 | MLP like protein 43 | 2 | 0.53 | 0.05 | Cytoplasm | C1-metabolism | N |
| 1. Glyma20g38740.1 | 10 formyltetrahydrofolate synthetase | 5 | 0.53 | 0.00 | Chloroplast | Stress | N |
| 1. Glyma07g13900.1 | Hyaluronan | 6 | 0.52 | 0.02 | Nucleus | RNA | N |
| 1. Glyma07g38790.1 | NAD(P) binding Rossmann fold superfamily protein | 5 | 0.51 | 0.04 | Nucleus | Misc | N |
| 1. Glyma09g25830.2 | CAP160 protein | 11 | 0.5 | 0.03 | Nucleus | Not assigned | N |
| 1. Glyma08g04490.1 | actin related protein 4 | 2 | 0.49 | 0.02 | Cytoplasm | Cell | N |
| 1. Glyma05g04290.1 | Glycosyl hydrolases family 32 protein | 8 | 0.46 | 0.00 | Secreted pathway | Major CHO metabolism | N |
| 1. Glyma14g10670.1 | KH domain containing protein | 8 | 0.45 | 0.01 | Nucleus | Protein.synthesis | N |
| 1. Glyma13g39790.1 | ABC transporter family protein | 2 | 0.45 | 0.02 | Cytoplasm | RNA | N |
| 1. Glyma13g21291.1 | embryonic cell protein 63 | 4 | 0.43 | 0.04 | Nucleus | Development | N |
| 1. Glyma11g12480.1 | cold circadian rhythm and RNA binding 2 | 3 | 0.42 | 0.04 | Chloroplast | RNA | N |
| 1. Glyma11g12490.1 | cold circadian rhythm and RNA binding 2 | 3 | 0.42 | 0.04 | Chloroplast | RNA | N |
| 1. Glyma03g27030.1 | DNAJ homologue 2 | 2 | 0.39 | 0.00 | Nucleus | Stress | N |
| 1. Glyma15g21890.1 | S adenosylmethionine synthetase family protein | 12 | 0.31 | 0.03 | Nucleus | Metal handling | N |
| 1. Glyma11g27480.1 | glutamine dependent asparagine synthase 1 | 6 | 0.28 | 0.03 | Cytoplasm | Amino acid metabolism | N |
| 1. Glyma11g27720.1 | glutamine dependent asparagine synthase 1 | 6 | 0.28 | 0.03 | Cytoplasm | Amino acid metabolism | N |
| 1. Glyma18g06840.2 | glutamine dependent asparagine synthase 1 | 6 | 0.28 | 0.03 | Cytoplasm | Amino acid metabolism | N |
| 1. Glyma10g07410.1 | embryonic cell protein 63 | 6 | 0.23 | 0.01 | Nucleus | Development | N |
| 1. Glyma12g32160.1 | Peroxidase superfamily protein | 2 | 0.22 | 0.03 | Secreted pathway | Stress.abiotic | Y |
| 1. Glyma12g32170.1 | Peroxidase superfamily protein | 2 | 0.22 | 0.03 | Secreted pathway | Misc | Y |
| 1. Glyma13g38300.1 | Peroxidase superfamily protein | 2 | 0.22 | 0.03 | Secreted pathway | Misc | Y |
| 1. Glyma13g38310.2 | Peroxidase superfamily protein | 2 | 0.22 | 0.03 | Secreted pathway | Stress.abiotic | Y |
| 1. Glyma08g13130.1 | evolutionarily conserved C terminal region 2 | 6 | 0.19 | 0.01 | Cytoplasm | Signalling.calcium | N |
| 1. Glyma05g30020.1 | evolutionarily conserved C terminal region 2 | 6 | 0.09 | 0.02 | Cytoplasm | Signalling.calcium | N |
| 1. Glyma11g10790.1 | nucleolin like 2 | 8 | 0.03 | 0.01 | Nucleus | Protein.synthesis | N |

Protein ID, according to the Phytozome database; M.P., matched peptide; Ratio, relative abundance of a protein from 4 day old soybean compared to 2-days old soybean root; ND, no description; Secretory Pathway, signal peptide presence based on the SignalP 4.1 Server.
