# Supplementary figures and images for: Quantitative proteomics reveals the effect of protein glycosylation in soybean root under flooding stress
Source: Front Plant Sci. 2014 Nov 18;5:627. doi: 10.3389/fpls.2014.00627 (PMC4235293; doi:10.3389/fpls.2014.00627)

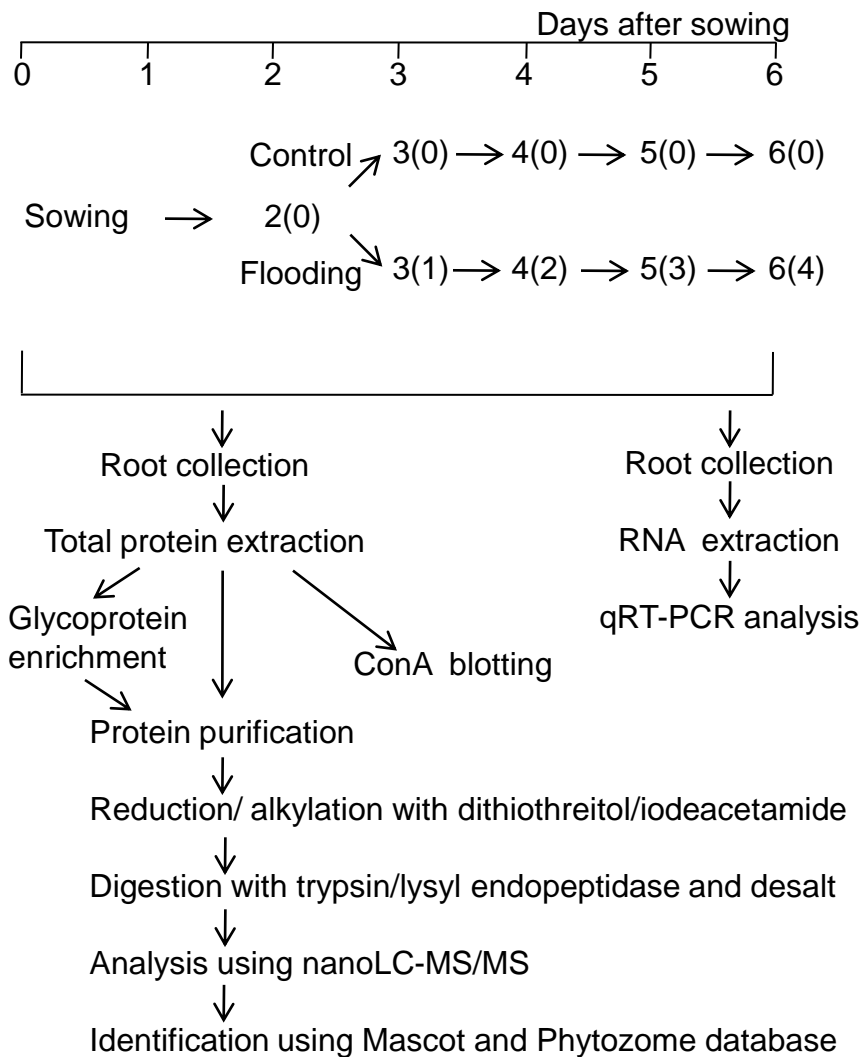

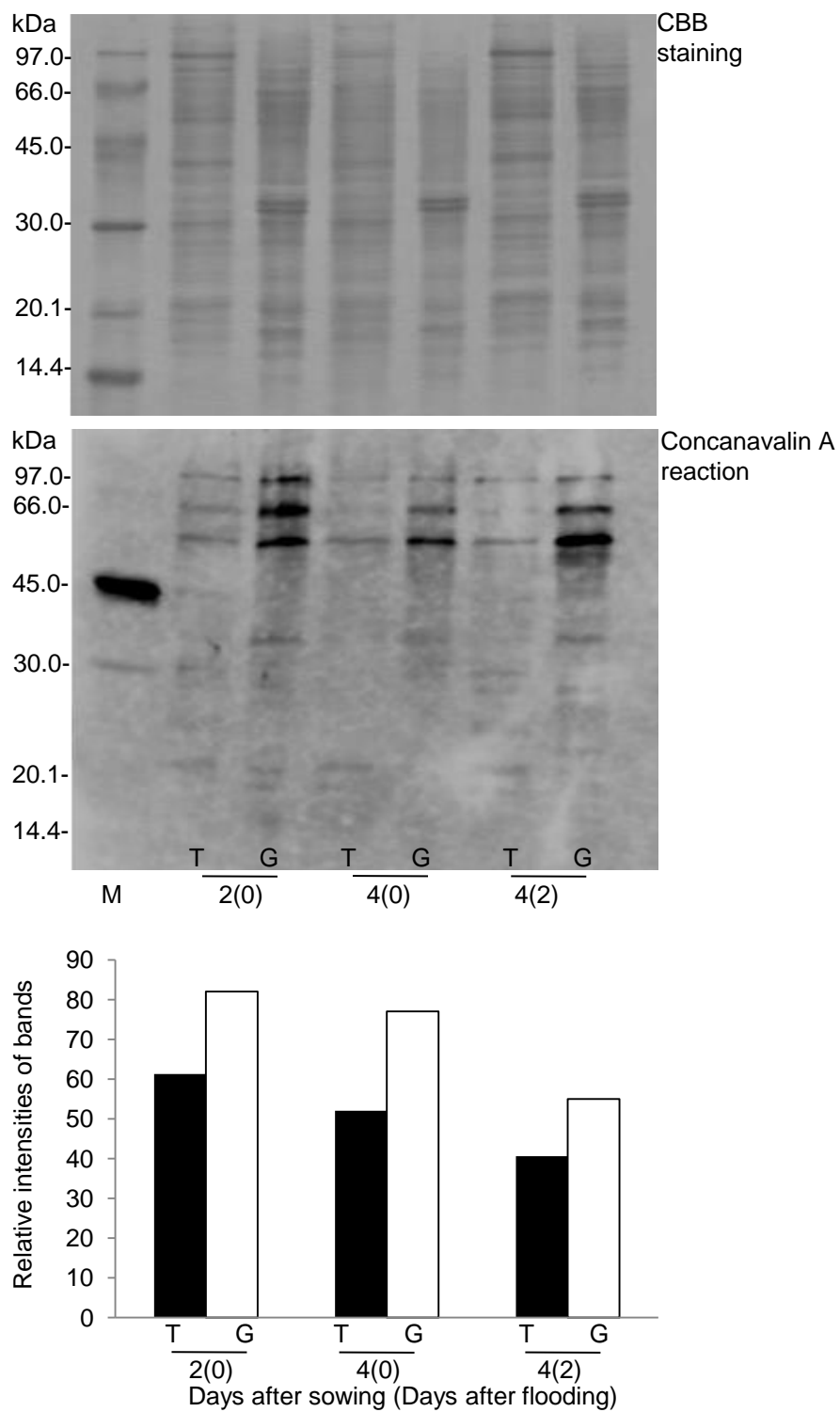

Supplemental figure 2

Supplement: Supplementary file 2 [file Image1.PDF]
